# Video laryngoscopy as a teaching tool in direct intubation in undergraduate medical education – a randomized controlled trial

**DOI:** 10.1186/s12909-026-08889-2

**Published:** 2026-02-24

**Authors:** Daniel Uhing, Lina Vogt, Martin Klasen, Michelle Schmidt, Maurice Elissen, Nico Haehn, Sasa Sopka, Michael Tobias Schauwinhold

**Affiliations:** 1 AIXTRA - Competence Center for Training and Patient Safety, Medical Faculty, RWTH Aachen, Aachen, Germany; 2 Department of Anaesthesiology, Medical Faculty, Uniklinik RWTH Aachen, RWTH Aachen, Pauwelsstraße 30, Aachen, 52074 Germany

**Keywords:** Airway management, Medical education, Skill training, Teaching-tool, Video-laryngoscopy

## Abstract

**Background:**

Direct laryngoscopy (DL) is a crucial skill and continues to be indispensable. While the superiority of video laryngoscopy (VL) has been demonstrated regarding the rate of first attempt successful intubations, its application as a teaching tool for DL remains largely unexplored. We hypothesised a higher first-pass success and shorter durations of conventional intubation after video laryngoscopy supported training (VLF = video-laryngoscopy based feedback) compared to students trained without VL (DLF = direct-laryngoscopy based feedback).

**Methods:**

All 211 medical students (DLF: 92/ VLF:119) trained conventional intubation, using a manikin in an airway course during their anaesthesiology rotation in fourth year of medical school. The study period was October 2023 to August 2024. In the DLF group, the tutor could only provide feedback via an intermittent direct view of the larynx during intubation whereas in the VLF group, the tutor had a continuous view via the monitor. Students of the VLF group did not get a view of the monitor and performed laryngoscopy via direct view. The main outcome measures were the first-pass success, time for intubation, Cormack-Lehane Score, occurrence of tooth damage, a difficulty score and a self-confidence score.

**Results:**

Both groups achieved a high rate of first-pass success (DLF 97,8% vs. VLF 100%, *p* = 0.081). Nevertheless, we found a significant difference concerning duration of intubation (DLF 21,49s ± 7,421 vs. VLF 24,75s ± 10,371, *p* = 0.008). DLF group demonstrated a higher reduction in a subjective difficulty score (*p* = 0.018). Both groups demonstrated enhanced confidence regarding airway management following training favouring DLF group (difference between the groups: *p* = 0.039).

**Conclusions:**

Teaching direct laryngoscopy by using the video laryngoscope monitor as a teaching tool for the tutor seems to give students more detailed feedback and a more realistic rating of the difficulty level, which is reflected in the students spending more time on the task, a smaller decrease in the difficulty value and a more realistic self-assessment. Furthermore, a higher first-pass success in our assessment with direct laryngoscopy is reached after video-laryngoscopy based feedback training. We assume that this method holds great chances for teaching practice.

**Trial registration:**

DRKS00032815 https://drks.de/search/de/trial/DRKS00032815.

## Background

Endotracheal intubation is a core skill in the field of anaesthesiology. Unfortunately, conventional intubation is difficult to learn and teach. Bernhard et al. demonstrated in 2011 that the first-pass success of a novice increased from 67% after the first 25 intubation attempts to 83% after 200 attempts [1]. The German guideline for prehospital airway management recommends 100 supervised endotracheal intubations and a repetition of 10 intubations per year as a minimum standard for personnel performing prehospital intubation attempts. These recommendations consider that difficult intubation occurs more frequently in prehospital settings [2]. A meta-analysis stated that novices need more than 50 intubations to reach a success rate of at least 90% in two intubation attempts under elective circumstances [3].

The introduction of VL as a tool for endotracheal intubation has led to significant improvements in airway management. Various large studies have shown the superiority of VL with respect to the rate of successful first-pass intubations [4–7]. Especially for patients with a predicted difficult airway situation, in obstetric aesthesia, for children with a difficult airway situation, neonates and emergency trauma patients VL was proven to be superior to DL [7–11]. A Cochrane review in 2016 with 7044 patients enclosing more than 64 studies identified VL reduced the number of failed intubations, especially for patients with a difficult airway situation. However, VL did not reduce the number of intubation attempts, hypoxia or other respiratory complications in this meta-analysis. Even the time required for intubation did not change between the two groups [12]. Other studies did not prove a higher first pass success e.g. a meta-analysis which included nine RCT with more than 2.000 ICU patients [13]. Even after the introduction of VL, DL is still considered an essential skill, such as in emergency situations with limited immediate availability of VL, technical issues like low battery power, or contamination of the camera by blood, fog, vomitus or other secretions [14]. If a video laryngoscope is available and already in use, heavy bleeding or vomiting can lead to contamination, causing the video component to become unusable. In these circumstances, even with a video laryngoscope in hand, it is necessary to resort to direct laryngoscopy skills, as described by other authors [15]. Whereas VL provides a better glottic view and may reduce laryngeal trauma, some studies described a higher risk of injuries of glottis wall structures, especially the right tonsillar pillars [12, 16]. The German guideline for airway management recommends the use of a video laryngoscope for rapid sequence inductions, emergency situations, patients with difficult airway and for critical ill patients. There is no general recommendation for the use of a video laryngoscope in the guideline [17]. Moreover, recent studies recommended that DL should be still taught and performed [14].

Even though a great amount of research investigated VL in clinical practice, the benefits of its use for teaching practice has hardly been studied. Commonly, the teaching process of direct laryngoscopy is hindered by the fact that the tutor and trainee cannot have a shared direct view of the larynx during the intubation process, which limits the possibility of supportive feedback. That is the advantage of VL, enabling the tutor to provide suggestions for improvement by observing the video laryngoscope monitor while the trainee uses a direct view for the intubation procedure. However, only few studies have investigated the use of VL as a teaching tool for learning conventional intubation in novices. A recent study examined the difference between two groups of a total of thirty-seven medical students after teaching intubation in two different ways. The first group received a training with VL, but only the teacher had view on the video laryngoscope monitor. The other group trained laryngoscopy with VL and shared the view with their teachers [18]. Another study regarding the use of VL as a teaching tool was performed in 2008 among forty-nine paramedics and medical students. They allowed their VL group the shared view on the video laryngoscope monitor for student and teacher. The other group performed their training with a standard Macintosh laryngoscope [19]. A further study using VL as a teaching tool was performed 2010. The authors recruited forty-two medical students without any intubation experiences and randomized their teaching into a VL group (GlideScope^®^) and a standard group (Macintosh laryngoscope). The VL group was allowed to use the video laryngoscope monitor for their training. The assessment was performed on real patients in general anaesthesia with a direct Macintosh laryngoscope [20]. The guideline for airway management in neonates and infants from the airway guidelines groups of the European Society of Anaesthesiology and Intensive Care (ESAIC) and the British Journal of Anaesthesia (BJA) considered the use of VL for teaching with a direct view for the trainee and view on the VL monitor for the tutor in a clinical practical statement [21]. However, further studies for this teaching approach especially in adult patients are missing.

Companies have launched video laryngoscopes to facilitate training in direct laryngoscopy. One example is the GlideScope Direct, which was designed specifically for this purpose [22]. However, studies investigating this teaching method are lacking, particularly among undergraduate students. The present study aims to address this issue.

In summary, our here presented study is the first study known which examined the use of VL monitor purely as a teaching tool for the tutor and compared this method with training by standard conventional laryngoscope.

We hypothesised a higher first-pass success and shorter durations of conventional intubation after VL supported training compared to students training without VL. Furthermore, we were interested in the differences regarding the time for the intubation procedure, the amount of tooth damage, the best Cormack-Lehane score achieved, a difficulty score and the sense of subjective safety.

## Methods

This manuscript adheres to the applicable CONSORT guidelines [23].

The study included fourth-year medical students at RWTH (Rheinisch-Westfälische Technische Hochschule) Aachen University with no previous experience in endotracheal intubation. As part of their anaesthesiology rotation, they received airway training by an experienced anaesthesiologist with at least four years of clinical experience. The participants were randomized into training with video-laryngoscopy based feedback (VLF) or direct-laryngoscopy based feedback (DLF). In an online pre-evaluation questionnaire conducted immediately before the start of the course, participants were asked demographic data, previous medical work experience as nurse or paramedic, how difficult they consider endotracheal intubation to be (difficulty score) and how confident they feel regarding intubation (subjective confidence). For the difficulty score a value of 1 was defined as very easy and 10 as impossible. Regarding the feeling of confidence, a value of 1 was defined as very unconfident and 6 as very confident. The training first consisted of a short introduction as a lecture regarding indication, procedure and the Cormack-Lehane classification. Afterwards, endotracheal intubation was taught using the Peyton 4-step approach [24].

**Fig. 1:**
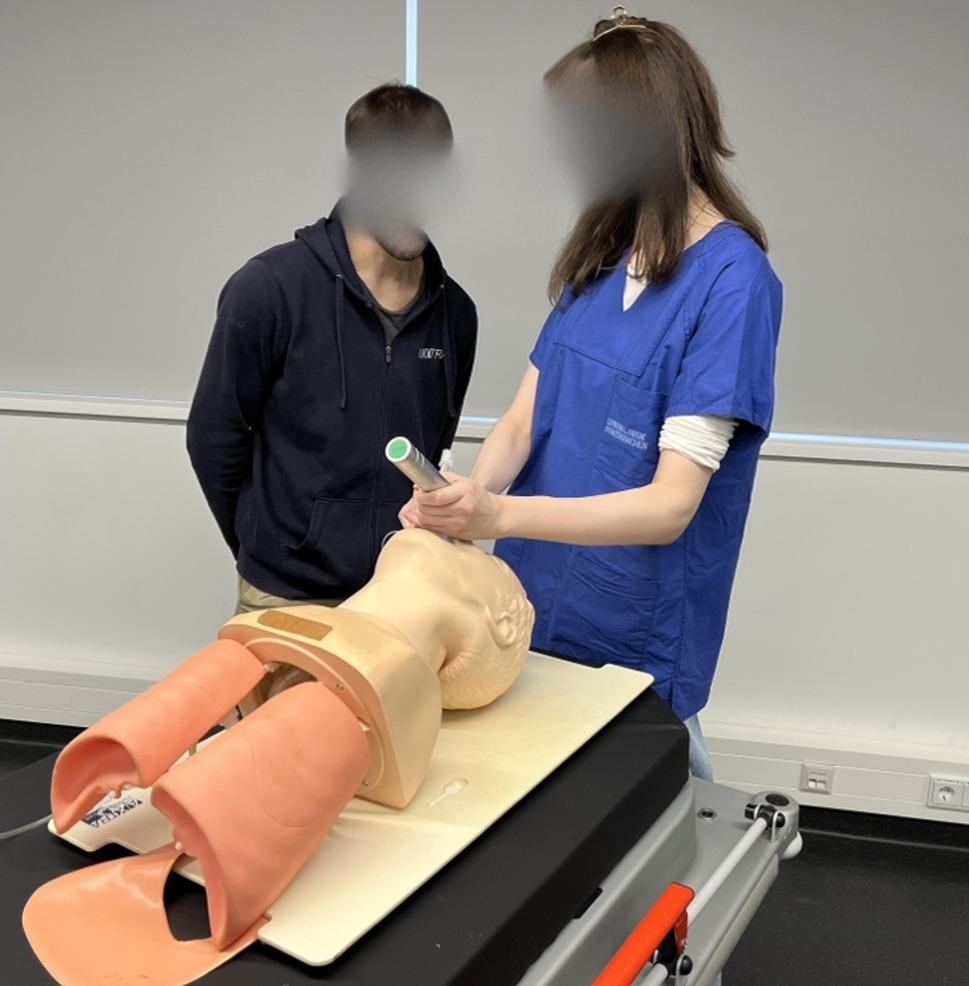
DLF group: student with direct view, tutor with intermitted direct view

**Fig. 2:**
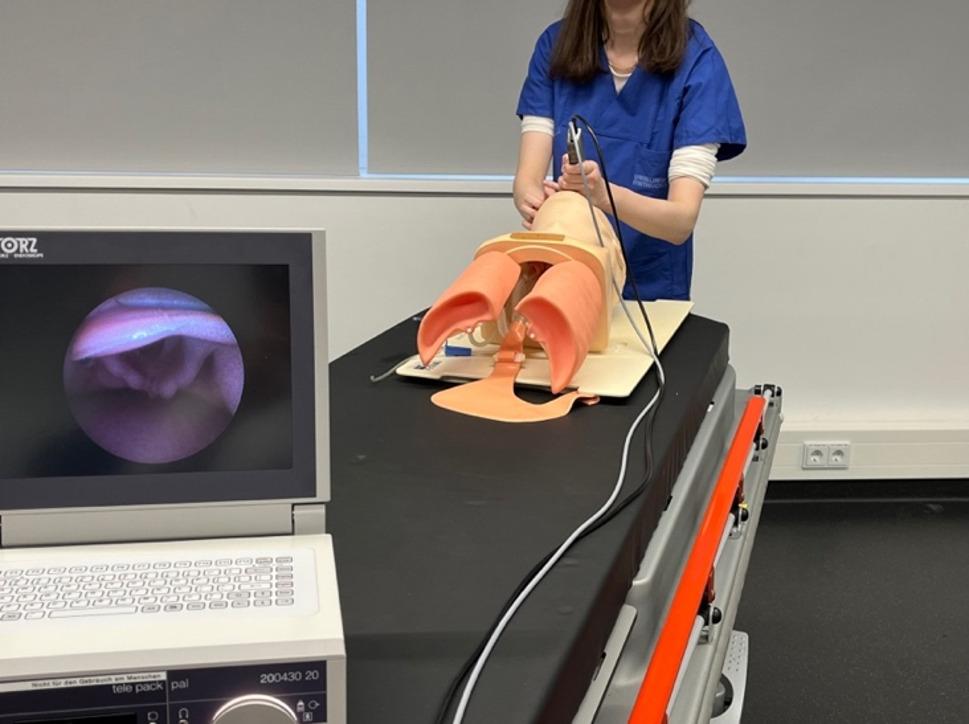
VLF group: student with direct view, tutor with view on the monitor

All participants (*n* = 211) trained conventional intubation, using a manikin (Laerdal^®^ Airway Management Trainer). In the DLF group (standard laryngoscope with Macintosh blade size 3), the tutor could only provide feedback via an intermittent direct view of the larynx during intubation (Fig. 1) whereas in the VLF-group (Karl Storz C-MAC Berci-Kaplan DCI Video Laryngoscope^®^ with a Macintosh blade size 3), the tutor was provided a continuous view via the monitor, while the trainee had no view of the monitor (Fig. 2).

The tutor gave solely verbal suggestions for improvement, while manual intervention by the tutor was not permitted. Each student in turn received a total of three training attempts. The other students were allowed to observe the training but did not intervene. The training trials began with bag-mask-ventilation on the airway model. On command by the tutor, endotracheal intubation began with hyperextension of the head, opening of the mouth and presentation of the laryngoscope. After successful visualisation of the larynx, the tube was offered by the assistant. After insertion of the tube into the trachea by the trainee, the assistant inflated the cuff on command. After each attempt, the correct ventilation of both lungs was examined using a resuscitation bag. Tooth damage was indicated by the airway model via a loud noise when too much pressure was applied to the upper row of teeth. An intubation stylet was not used.

Afterwards, a standardized assessment took place using the same airway trainer. The procedure corresponded to the training, with assistance being given by a trained member of the study team. The tutor did not provide any feedback. The assessment ended after one successful intubation attempt or a maximum duration of 120 s.

After the airway course, a post-evaluation questionnaire was conducted. Hereby, again, the difficulty score and the subjective confidence of the study participants were assessed.

### Primary outcome parameters

1. Rate of first pass -success (tube tip below the glottis in the trachea).

### Secondary outcome parameters

1. Time from acceptance of the laryngoscope to command to inflate the cuff.
2. Tooth damage.
3. Best view according to Cormack-Lehane score (printout was available in the assessment room).
4. Difficulty score (1–10).
5. Subjective confidence (1–6).

Sample size planning for the primary outcome parameter (Chi² goodness-of-fit-test for contingency tables) was carried out in advance using the software G*Power 3.1.9.7 and resulted in a total sample size of 108 study participants, assuming a medium effect size w(φ) of 0.3, an alpha error probability of 0.05, and an assumed power of 0.80. Students were randomly allocated, ranging from October 2023 to August 2024. Subsequently, the time points were block-randomized into two conditions (VLF and DLF) using the software Research Randomizer (https://www.randomizer.org/), with equal proportions for both conditions.

### Statistical analysis

Data were analysed with IBM SPSS Statistics Version 29.0.0.0 (IBM Corp., Armonk, NY, USA). The primary outcome parameter (rate of first-pass success) as well as the secondary outcome parameter “tooth damage” were analysed using Chi² tests. The secondary outcome parameters “time from acceptance of the laryngoscope to command to inflate the cuff” and “best view according to Cormack-Lehane score” were analysed with t-tests for independent samples. Finally, difficulty and subjective confidence scores were analysed in two-way mixed model analyses of variance (ANOVA), with the within-subjects factor time point (pre-training/post-training) and the between-subjects factor group (VLF/DLF). For all tests, significance was determined as *p* < 0.05.

## Results

A total of 211 fourth-year medical students participated in the described airway training course during their anaesthesiology internship. The study took place from October 2023 to August 2024. The demographic data are presented in Table 1 (Table 1).

**Table 1:** Sample demographics

Gender	male	female	diverse
	34,3%	65,2%	0,5%
Age	minimum	maximum	Mean and SD
	20	37	24,19 ± 3,212
Previous experience	emergency service	nursing	none
	6,7%	14,8%	78,5%

Table 2 shows the results of our study (Table 2). The success rates of endotracheal intubation, as previously defined, were markedly high in both groups. The DLF group achieved a first-pass success rate of 96.7%, while the VLF group demonstrated an even higher success rate of 100% in the assessment, with no significant difference observed (Chi²(2) = 3.94; *p* = 0.081).

**Table 2:** Results

	DLF-group	VLF-group	*p*-value
	*n* = 92	*n* = 119	
PRIMARY OUTCOME			
First pass success (absolute frequency and percentage)	89 (96.7%)	119 (100%)	0.081
SECONDARY OUTCOMES			
Time for intubation (seconds, mean ± SD)	21.49 ± 7,421	24.75 ± 10.371	**0.008**
Cormack-Lehane Score (mean ± SD)	1.61 ± 0.49	1.58 ± 0.51	0.680
Tooth damage (absolute frequency and percentage)	6 (6,52%)	11 (10.18%)	0.612
Reduction in perceived intubation difficulty (scale from 1–10, mean ± SD)	3.05 ± 1.61	2.5 ± 1.60	**0.018**
Enhanced confidence in intubation (scale from 1–6, mean ± SD)	2.17 ± 1.12	1.82 ± 1.05	**0.039**

Significant results (*p*<0.05) are marked in bold

A total of only two instances of oesophageal malposition were observed throughout the entire study period. Both instances occurred in the DLF group. Furthermore, the endotracheal tube was placed at an inappropriate depth on one occasion. This occurred only in the DLF group as well. The very low frequency of unsuccessful intubations led to an expected *n* < 5 in several cells; since the Chi² does not deliver reliable results at such small expected frequencies, we controlled the results of the contingency table furthermore with Fisher‘s exact test, leading to a similarly non-significant result (*p* = 0.081).

The DLF group required 21.49 s ± 7.421 to complete the task, which was significantly shorter than the VLF group which required 24.75 ± 10.371 s (T(208) = 2.66; *p* = 0.008).

In the pre-evaluation the participants in total rated endotracheal intubation with a difficulty score of 6.83 ± 1.801. The DLF group had a score of 6.89 ± 1.748 whereas the VLF group had a score of 6.79 ± 1,845. Following the airway training, both groups demonstrated a notable reduction in the difficulty score, towards a score of 3,84 ± 1.485 in the DLF group and 4.29 ± 1.656 in the VLF group. A 2 × 2 analysis of variance (ANOVA) showed a significant interaction of group x time point (F(1,202) = 5.72; *p* = 0.018), indicating a stronger reduction of the difficulty score in the DLF group.

Prior to the training, the mean score for confidence in performing endotracheal intubation was 2.36 ± 1.215 (DLF group: 2.19 ± 1.114/ VLF group: 2.49 ± 1.275). Both groups demonstrated an enhanced sense of safety regarding airway management following the training, with a mean score of 4.36 ± 0.888 for the DLF group and 4.31 ± 0.873 for the VLF group. A 2 × 2 analysis of variance (ANOVA) showed a significant interaction of group x time point (F(1,171) = 4.31; *p* = 0.039), indicating a stronger increase of safety regarding airway management in the DLF group.

## Discussion

Our study results did not support our hypothesis. The DLF-group, who received training without the possibility of direct tutor feedback via the video laryngoscope monitor, achieved a comparably high first-pass success rate and performed faster in terms of time to intubation, had more decrease in difficulty and a greater increase in subjective confidence. There were no significant differences regarding the Cormack-Lehane score achieved or the tooth damage.

While there are numerous studies that describe the superiority of VL for endotracheal intubation (higher first-pass-success, visualisation of the glottis, faster intubation time, less additional required manoeuvres), especially in the case of difficult airway situations, the literature on VL used solely as a teaching tool for direct laryngoscopy is sparse [5–7].

Learning endotracheal intubation follows a typical learning curve as described in a previous study [1]. Another investigation stated a success of 60% after 20 and 90% after 80 intubations [25]. A recent paper recommends a minimum of 119 endotracheal intubations to achieve a first-attempt success from ≥ 85% in the emergency department [26]. The high success rates of both groups in our study (DLF: 97.8%, VLF: 100%) after only three training attempts deviate from the usual learning curve for endotracheal intubation. One reason for this could be that intubation of the manikin was too easy compared to intubation of real patients. This may lead to a ceiling effect, which in turn concealed the possible benefits of VLF in our study. A study from Yi et al. compared the teaching of medical students with DL using a McGrath video laryngoscope whereby only the tutor had a monitor view for feedback versus an indirect group where student and tutor used the McGrath video laryngoscope monitor (shared view). Both groups showed 100% first pass success in an assessment in the Laerdal^®^ Airway Trainer with a direct Macintosh laryngoscope after five training attempts [18]. This study supports our assumption that the Laerdal^®^ Airway Management Trainer seems to be too easy for learning purposes which can lead to a ceiling effect.

A study by Sakles et al. showed that adverse events like aspiration, oxygen desaturation, esophageal intubation, hypotension and others significantly increase as the number of intubation attempts increases. The retrospective study examined more than 1,800 intubations in an emergency department over a period of four years. The rate of adverse events tripled when a second attempt was needed. After four and more attempts, the incidence of adverse advents was more than 70% [27]. The link between multiple intubation attempts and adverse events has also been confirmed in other studies [28, 29]. Therefore, a first pass success rate as high as possible is desirable. In our study three students of DLF group failed endotracheal intubation in the first attempt whereas every intubation attempt of our VLF group was successful. This result may be explained by more detailed feedback during training in the VLF group and emphasises the advantage of this training method.

We measured the time from acceptance of the laryngoscope to command to inflate the cuff. Participants of both groups were able to secure the airway within a short period of time 21,49 s in the DLF group and 24,75 s in the VLF group. In a study described by Herbstreit et al., the times achieved after training (between acceptance of the laryngoscope and passage of the tube through the glottis) were also between 18 and 29 s [30]. In our study we measured the time from the acceptance of the laryngoscope till the command to inflate the cuff, which may extend the time needed. Another study compared DL and VL in 1000 patients undergoing general anaesthesia with the need for endotracheal intubation performed by trained anaesthesiologists. The time from device insertion to endotracheal placement were 20 s for the VL group (view of the monitor was allowed) and 27 s for the DL group, which generally is comparable to our results [31]. We assume that the time difference between the groups of around 3 s in our study is unlikely to have a great clinical impact.

Lifting the epiglottis with the blade is a typical beginner’s mistake. However, it is possible that this error was not regularly recognized and corrected in the DLF-group, as continuous supervision was not possible in this group. Successful intubation is often still possible despite lifting of the epiglottis. This could have led to faster times in the DLF-group. Further studies must identify which aspects in detail lead to more needed time for intubation.

The reported decrease in the difficulty score is interesting. As expected, this decreased in both groups after the training. However, it decreased even more in the DLF group than in the VLF group. We assume that the video-laryngoscopy based feedback enabled the tutor to provide much more detailed feedback. A common feedback strategy is the direct observation which we used during and after every of the three training attempts in our study. This strategy is known for improving performance in students, especially when the feedback occurs immediately after the intervention, as described recently [32]. The more detailed feedback, possible through the teachers view on the video laryngoscope monitor in the VLF group, may have made the participants more aware that their intubation attempts needed to be improved even further than was necessary for the insertion of the endotracheal tube. The longer time required for the assessment may also be explained by the fact that the participants, who had previously received feedback from the tutor with a view of the video laryngoscope monitor, tried to further improve the intubation conditions, although intubation might already have been possible. A stronger improvement in the enhanced confidence of the measure in the DLF group, as shown in our results, could also be due to this effect.

Limitations and further work:

We used a manikin for our training and assessment which can differ from normal intubations in real patients. It might be possible, that some participants of the study had some prior experience in endotracheal intubation. On the other hand, only a minority of the participants had received prior training in the medical field, including emergency medical services and nursing training. However, as these medical educations do not encompass endotracheal intubation, it can be assumed that all participants were novices in performing this procedure.

The shape of the handle feels slightly different between the video laryngoscope handle and the conventional laryngoscope. Even though the same handle was used during the data collection and the assessment, the shape could have caused a difference between the groups. Therefore, further studies could investigate the influence of the haptics of the laryngoscope in greater detail.

During the training, the other study participants were able to observe their colleagues’ intubation attempts. While doing so, they could not see the larynx, either in the DLF group nor via the video laryngoscope monitor in the VLF group. The VL monitor was only accessible to the tutor. For this reason, we do not believe that the other students gained any significant advantage for their own intubation attempts. At most, they could observe the opening of the mouth and the handling of the laryngoscope from the outside. As the study procedure was identical in both groups, this should not have had a significant impact on the results.

## Conclusion

We assume that teaching direct laryngoscopy by using the video-laryngoscopy based feedback as a teaching tool holds great chances for teaching practice. This method seems to give students more detailed feedback and a more realistic rating of the difficulty level, which is reflected in the students spending more time on the task and a more realistic self-assessment. Studies using more challenging manikins may also show superiority in first-pass success, as this study was possibly limited by an assumed ceiling effect.

## Data Availability

The datasets used and analysed during the current study are available from the corresponding author on reasonable request.

## Abbreviations

ANOVA: Analysis of Variance
BJA: British Journal of Anaesthesia
DL: Direct Laryngoscopy
DLF: direct-laryngoscopy based feedback
ESAIC: European Society of Anaesthesiology and Intensive Care
ICU: Intensive Care Unit
RWTH: Rheinisch-Westfälische Technische Hochschule
VL: Video Laryngoscopy
VLF: video-laryngoscopy based feedback
